# Subglottic Stenosis as a Presentation of Antineutrophil Cytoplasmic Antibody-Associated Glomerulonephritis

**DOI:** 10.7759/cureus.46899

**Published:** 2023-10-12

**Authors:** Austin Patrick Eisenberg, Victor Collier, Andrew Mangano, Karandeep Shergill, Ellen Yos

**Affiliations:** 1 Internal Medicine, Grand Strand Medical Center, Myrtle Beach, USA; 2 Internal Medicine, Mary Washington Healthcare, Fredericksburg, USA; 3 Nephrology, Grand Strand Medical Center, Myrtle Beach, USA

**Keywords:** pauci-immune glomerulonephritis (gn), anca associated vasculitis, glomerulonephritis (gn), chronic kidney disease (ckd), polyangiitis, granulmatosis, subglottic stenosis

## Abstract

Granulomatosis with polyangiitis (GPA), previously Wegener’s granulomatosis, is a necrotizing vasculitic disease process affecting the small- and medium-sized blood vessels. GPA is frequently associated with damage to the respiratory tract and kidneys but often affects other organ systems including the eyes, nasopharynx, and nervous system. Due to the vague nature of presenting symptoms and the progressive nature of GPA, it is essential to keep a broad differential to mitigate the high morbidity and mortality associated with the disease. Here we introduce a case of a GPA presenting as respiratory distress, stridor, and renal injury. We also review common clinical presentations, diagnostic evaluation, and treatment options.

## Introduction

Granulomatosis with polyangiitis (GPA), previously known as Wegener’s granulomatosis, is one of the three antineutrophil cytoplasmic antibody (ANCA)-associated vasculitides [1]. GPA causes necrotizing granulomatous inflammation in the upper and lower respiratory tract, necrotizing vasculitis of the small and medium blood vessels, and necrotizing glomerulonephritis. GPA can also present with other organ system involvement, such as ophthalmologic, neurologic, and skin manifestations [1-3]. The diagnosis of GPA is made through a constellation of clinical and laboratory findings such as histopathology showing granulomatous inflammation, presence of ANCA antibodies, abnormal imaging of the chest, urinalysis showing sediment with red blood cells, and oral or nasal inflammation [4].

The most common manifestations of GPA are respiratory tract involvement, both upper and lower, and kidney involvement. Patients with respiratory involvement often present with chronic cough, wheezing, hemoptysis, epistaxis, and rhinitis [5]. Pulmonary radiologic findings commonly include multiple nodules of various sizes in a random distribution, which can be cavitary lesions, pleural effusions, as well as ground glass opacities/consolidations in the setting of alveolar hemorrhage [6]. Renal manifestations often include hematuria, proteinuria, and reduced glomerular filtration rate (GMR). Radiologic findings are not typical for renal involvement, though histopathology commonly shows pauci-immune glomerulonephritis [2,5].

The pathophysiologic mechanism of GPA is not fully understood. However, this autoimmune condition may arise due to the overactivation of B and T cells, leading to endothelial damage [7]. It follows that the treatment for GPA typically involves immunosuppressive agents such as cyclophosphamide, methotrexate, and rituximab, as well as glucocorticoids, to aid in reducing inflammation [8,9].

## Case presentation

Our patient was a 68-year-old female with a past medical history of insulin-dependent diabetes mellitus (IDDM) and chronic kidney disease stage 3b (CKD3b) who presented to our facility at the request of her otolaryngologist due to worsening dysphagia and stridor. The patient reported being in good health until approximately five months before admission to our hospital. At that time, she was diagnosed with community-acquired pneumonia and treated with seven days of doxycycline. Her symptoms improved for a short period but did not resolve. She was subsequently hospitalized at another outside hospital for community-acquired pneumonia two months before admission at our facility. 

**Figure 1:**
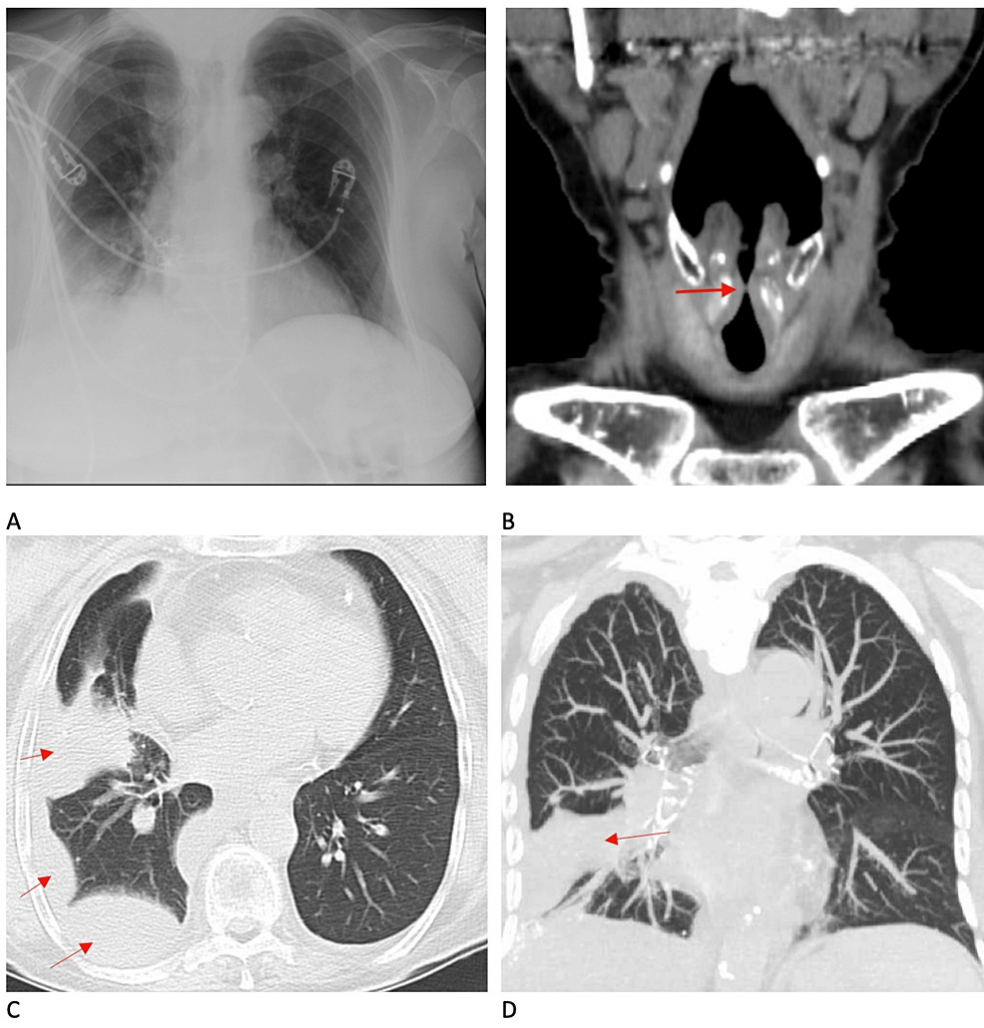
Imaging of the patient's chest and neck A. Chest X-ray demonstrating small right pleural effusion and adjacent atelectasis B. CT scan of the neck demonstrating subglottic stenosis below the level of the vocal cords (dark red arrow) C. CT scan of the chest, coronal view, demonstrating complex fluid collections in the right lung (red arrows) D. CT scan of the chest, axial view, demonstrating complex fluid collections in the right lung (red arrow)

Imaging of the patient's chest and neck was obtained via X-ray and CT. As seen in Figure 1A, the initial chest X-ray was remarkable for a small right pleural effusion and adjacent atelectasis. CT scans of her neck and chest were remarkable for tracheal stenosis below the level of the vocal cords (Figure 1B), as well as complex fluid collection in the right lung, small pulmonary nodules in the right upper lobe, and infiltrates in the right middle and lower lobes (Figures 1C, 1D). She also presented with kidney injury of unknown chronicity and etiology, with blood urea nitrogen (BUN) and creatinine 32 mg/dl and 2.0 mg/dl, respectively. GFR was estimated at 25-30 mL/min (normal > 60 mL/min). Her baseline BUN and creatinine prior to this hospitalization were unknown. Urinalysis showed proteinuria of 50 mg/dl (normal 0 mg/dl), and hematuria of 0.2 mg/dl (normal 0 mg/dl).

**Figure 2:**
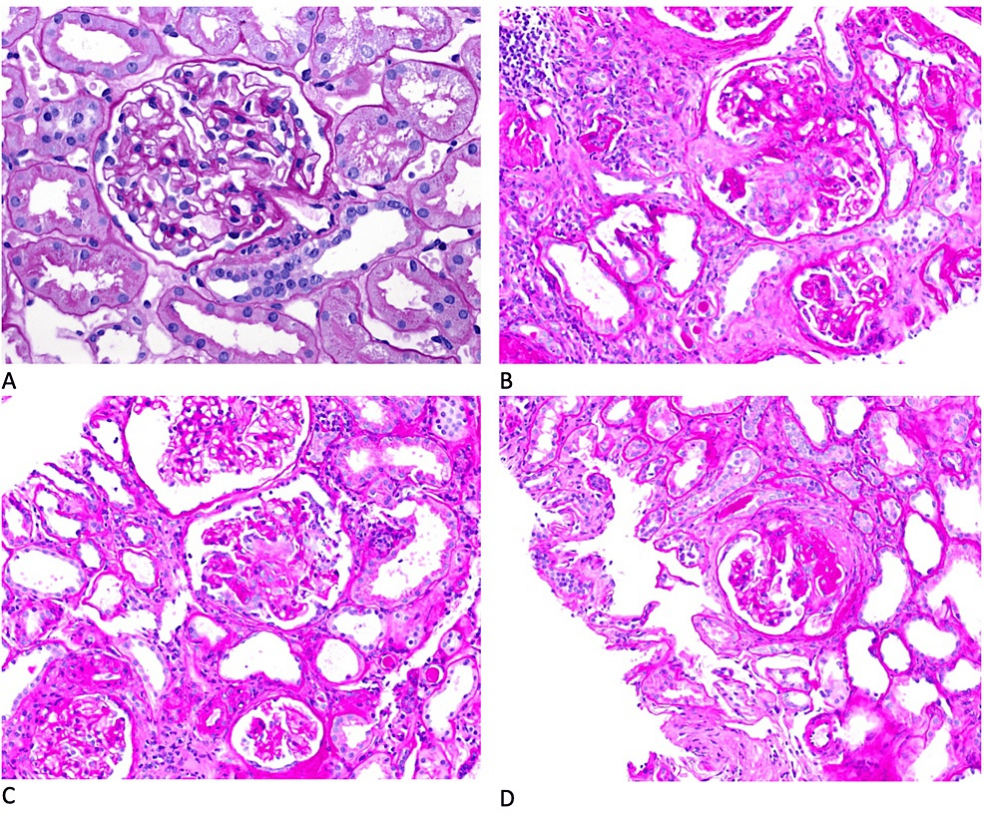
Kidney biopsies taken from our patient demonstrate a normal glomerulus (A), fibrocellular crescents (B), necrotizing lesions (C), and segmental sclerosis (D)

Table 1 demonstrates pertinent autoimmune and inflammatory lab markers that furthered our suspicion of GPA. Other autoimmune labs were negative, including antinuclear antibody (ANA), rheumatoid factor, SS-A/Ro, and SS-B/La. After discharge, a CT-guided kidney biopsy was obtained and sent for histopathology, which showed pauci-immune necrotizing and crescentic glomerulonephritis (Figure 2).

**Table 1:** Lab values ANCA: antineutrophil cytoplasmic antibody

Lab	Value
C-ANCA	6.8 units/mL (0.0-0.9 units/mL)
P-ANCA	2.0 units/mL (0.0-0.9 units/mL)
C-reactive protein	3.0 mg/dL (0-0.99 mg/dL)
Complement C3	130 mg/dL (80-167 mg/dL)
Complement C4	28 mg/dL (12-38 mg/dL)
Anti-glomerular basement membrane antibody	3 units (0-20 units).

Our team was suspicious of the diagnosis of ANCA vasculitis at discharge, but it had not been confirmed. She was sent home on 40 mg of daily prednisone and referred to outpatient Rheumatology. Rituximab therapy was initiated by her rheumatologist approximately one month after discharge. Since then, she has been closely followed by both a pulmonologist and a nephrologist. Unfortunately, her kidney disease progressed to a point where she required fistula placement and hemodialysis.

## Discussion

GPA is a rare albeit severe autoimmune phenomenon that leads to widespread necrotizing and granulomatous inflammation. GPA most commonly affects the respiratory tracts and the kidneys, but it can affect any organ system [1-3,5]. GPA is one of three ANCA-associated vasculitides, the other two being eosinophilic granulomatosis with polyangiitis and microscopic polyangiitis.

GPA affects roughly 2-11 people out of one million; peak symptom onset is 41-68 years old, more frequently affecting Caucasians from Northern Europe [10]. The pathophysiologic mechanism is not entirely understood, but the disease is believed to be driven by the overactivation of B cells and T cells. Specifically, imbalances in T cell subtypes lead to the breakdown of tolerance and an increased oxidative burst [7].

GPA is associated with autoantibodies against myeloperoxidase (MPO), also called P-ANCA, and autoantibodies against proteinase-3 (PR3), also called C-ANCA. A larger portion of GPA is the PR3 subtype compared MPO subtype [10-12]. The MPO subtype is associated with a worse prognosis and increased incidence of severe renal injury and alveolar hemorrhage [11]. Resistance to treatment is more common in the MPO subtype. The PR3 subtype has a better prognosis but tends to have more frequent relapses [11,12].

Due to its ability to affect nearly any organ system, GPA can present in multifarious ways. The most common initial manifestations are fever, weight loss, and malaise [5]. Our patient’s presenting complaint was respiratory distress, dysphagia, and stridor in the setting of recurrent pneumonia. Initially worked up for dysphagia and stridor. However, it was her renal disease, pleural effusions, and tracheal stenosis that led to further autoimmune workup and, subsequently, the pursuit of vasculitis. In an effort to lower long-term morbidity and mortality in these patients, clinicians must keep a broad differential and a low threshold to work up GPA. Chronic cough and pulmonary nodules seen on imaging should raise suspicion for GPA in the proper clinical context. Saddle nose deformity and septal perforation are common as well [8]. As was the case of our patient, subglottic stenosis presenting as stridor and respiratory distress is not an uncommon albeit distressing manifestation of the disease [13,14]. Ocular involvement (scleritis, episcleritis) and oral ulcers are common in GPA. Up to 15% of cases have ocular involvement at the time of presentation [15]. Glomerulonephritis develops in many patients with GPA, most commonly necrotizing glomerulonephritis (GN) [16]. As seen in figures 4.2-4.4, a kidney biopsy from our patient demonstrated necrotizing lesions, segmental sclerosis, and fibrocellular crescents, with a lack of positive immunofluorescence these findings suggested pauci-immune GN.

Diagnosis of GPA is multifactorial, based on clinical manifestations, findings on high-resolution chest CT, positive ANCA labs, and histopathology showing necrotizing granulomatous inflammation [1,4,8]. Bilateral lung nodules in subpleural regions on chest CT and a rim of ground glass opacities (if diffuse alveolar hemorrhage is present) also suggest GPA [17,18]. Other common imaging findings include pleural effusions and tracheal/upper respiratory tract thickening leading to stenosis, which were the impetuses for our patient’s admission. Renal pathology is infrequently seen on routine imaging (CT/MRI). However, roughly 10-20% of patients have renal involvement at the time of diagnosis, including proteinuria, hematuria, and renal failure, but up to 80% will develop renal involvement within 2 years of diagnosis [19]. The prognosis for patients with untreated GPA is poor, with mortality greater than 80% at 1 year post-diagnosis [1]. Thus, it is imperative that we as clinicians refrain from anchoring and keep a broad differential when patients present with kidney disease, both chronic and acute, and there is no obvious explanation. 

As the pathophysiology of GPA is driven by T and B cells, the mainstay of treatment involves immunosuppression, often a cytotoxic agent and a glucocorticoid [8,20]. Common cytotoxic agents include azathioprine, leflunomide, methotrexate, mycophenolate mofetil, and cyclophosphamide (CYC) [20]. Rituximab, a monoclonal antibody against CD-20, is a mainstay of GPA treatment [1]. Newer agents, including deoxyspergualin and abatacept, are becoming increasingly popular [21,22]. Cyclophosphamide or rituximab and a glucocorticoid are excellent options to treat severe life-threatening presentations, while less severe manifestations respond well to methotrexate or mycophenolate mofetil, plus a glucocorticoid [21]. Tracheal/subglottic stenosis responds well to intralesional glucocorticoid injections, Although our patient did not require such treatment [13,14]. Tracheal stenosis can also be treated with more invasive measures, a safer option being endoscopic balloon dilation [13,14]. Once symptoms are in remission, treatment involves rituximab, methotrexate, or mycophenolate mofetil in combination with a long glucocorticoid taper [20,21].

## Conclusions

Granulomatosis with polyangiitis (GPA) is a devastating autoimmune condition that primarily causes necrotizing granulomatous inflammation and vasculitis. GPA most commonly affects the respiratory tracts and kidneys but can involve any organ system, making the initial diagnosis a challenge. Our patient presented with stridor, respiratory distress, and renal injury of unknown chronicity and was diagnosed with GPA after discharge. The morbidity and mortality of untreated GPA are extremely high, making it important to keep a broad differential, avoid anchoring, and remain vigilant when patients present with an unclear constellation of symptoms.
